# CD40 Ligand Potentiates Immunogenecity of *Mycoplasma pneumoniae* Subunit Vaccine Candidate in a Murine Model

**DOI:** 10.3390/cimb47010037

**Published:** 2025-01-09

**Authors:** Jinqi Shu, Gaojian Li, Jianhong Shu, Huapeng Feng, Yulong He

**Affiliations:** 1 Department of Biopharmacy, College of Life Sciences and Medicine, Zhejiang Sci-Tech University, Hangzhou 310018, China; 201920201058@mails.zstu.edu.cn (J.S.); 201820201018@mails.zstu.edu.cn (G.L.); shujianhong@zstu.edu.cn (J.S.); fenghuapeng@zstu.edu.cn (H.F.); 2 Research Center of Animal Vaccines and Diagnostic Reagents, Zhejiang Sci-Tech University Shaoxing Academy of Biomedicine, Shaoxing 312090, China

**Keywords:** *Mycoplasma hyopneumoniae*, subunit vaccine, baculovirus expression system, molecular adjuvant, CD40L, immunoenhancement

## Abstract

*Mycoplasma hyopneumoniae* (Mhp) infection severely affects the daily weight gain and feed-to-meat ratio of pigs, while secondary infections with other pathogens can further lead to increased mortality, causing significant economic losses to the pig industry. CD40L is a molecular adjuvant that enhances the cellular and humoral immune responses to vaccines. In this study, the CD40L peptide was fused to the C-terminus of the chimeric P97R1P46P42 protein by genetic engineering using the pFastBac Dual vector. The recombinant chimeric protein P97R1P46P42 and its fusion P97R1P46P42-CD40L were expressed in Sf9 cells and purified. Mice were immunized with P97R1P46P42 or its fusion protein. Seppic ISA 201 emulsified protein, conventional Mhp vaccine and PBS control groups were included. Immunogenecity was assessed by specific IgG antibody response, splenic lymphocyte proliferation, and cytokine IL-4 and IFN-γ levels. We found that CD40L fusion significantly enhanced specific antibody response, lymphocyte proliferation and IL-4 level in the immunized mouse sera as compared to the P97R1P46P42 or conventional vaccine group. This study provides clear evidence that CD40L potentiates the humoral and cellular immune responses to the Mhp chimeric protein P97R1P46P42 in the mouse model. This CD40L-fused chimeric protein could be a MPS subunit vaccine candidate to be tested for its efficacy in pigs in response to challenges with pathogenic *Mycoplasma hyopneumoniae* strain(s).

## 1. Introduction

*Mycoplasma hyopneumoniae* (Mhp) is the primary pathogen responsible for mycoplasmal pneumonia of swine (MPS), often referred to as porcine enzootic pneumonia. MPS is a chronic respiratory disease worldwide and has a high prevalence in pigs from mid-fattening to slaughter [1]. The primary symptoms include persistent yet ineffective cough, decreased average daily weight gain, and low feed conversion ratio [2,3]. Mhp belongs to Gram-negative facultative anaerobic bacteria and colonizes the ciliated epithelium of the porcine respiratory tracts. Animals infected with Mhp are more prone to concurrent infections with other pathogenic bacteria, viruses and parasites [4,5]. Mhp infections cause significant losses to the swine industry [4,6,7,8].

The prerequisite for the induction of MPS is specific adhesion of Mhp to the cilia of porcine respiratory epithelial cells. This adhesion is dynamic and complex, encompassing the concerted action of multiple related functional adhesion factors (P159, P102, P97, and P95, among others), and both the pathogenicity and immunogenicity of Mhp are intimately linked to these adhesion factors on the bacterial membrane [2,9,10]. Among them, adhesin P97 is the main adhesion protein of Mhp by binding to host cell receptor molecules. The adhesion process is directly facilitated by P97R1, the C-terminal portion of P97 [11,12,13]. Under stressful situations, the HSP70 family member molecular chaperone DNAK (P42) is extensively expressed [14,15,16]. P46, a 46 kDa membrane surface protein, is specifically recognized by convalescent swine serum [15,17]. These proteins are good candidates for immunogens for vaccine development.

The most effective method of combating MPS is vaccination, as evidenced in North America and Europe [6]. To date, a few inactivated and attenuated vaccines have been approved for use, and they can help alleviate clinical symptoms and lung lesions of the pigs. [18,19,20,21]. Nonetheless, the precise mechanisms required to prevent Mhp infection remain poorly understood, and researchers are continually working to develop new vaccines that offer better protection [22,23].

Molecular adjuvants are single-molecule natural immune stimulators that differ from conventional adjuvants or liposomal nanoparticles in that they are plasmid-encoded proteins that enhance adaptive immune responses against antigens by targeting natural immune receptors or modulate molecular signaling [24,25,26]. Molecular adjuvants include pathogen-recognizing receptor agonists, cytokines, chemokines, and immune-targeting genes, among others. CD40 ligand (CD40L) has been used as a molecular adjuvant in numerous studies to improve the cellular and humoral immune responses to vaccinations [27,28,29]. The tumor necrosis factor superfamily includes the CD40 ligand (CD40L, CD154) which is expressed in endothelial cells, B cells, dendritic cells and macrophages. The 39 kDa type II transmembrane glycoprotein known as CD40L is located on the X chromosome [30] and is absent from resting naive T cells. In contrast, when T cells are activated, the transient expression of CD40L enables the T cells to induce isotype conversion to IgE. In addition, the interaction between CD40 and CD40L can effectively promote the proliferation and survival of antigen-presenting cells (APC), B cells, and T cells, and CD40-CD40L plays a crucial role in encouraging CD4T cells and dendritic cells to start producing cytotoxic T lymphocytes [31,32]. CD40L induces optimal T-cell activation and cytokine production [33].

Recombinant Mhp P97R1P46P42 chimeric proteins have been previously expressed in our laboratory using a prokaryotic expression system and an insect baculovirus surface display system [27,28]. The recombinant baculovirus P97R1P46P42 protein was effective in inducing humoral and cellular immune responses in mice and piglets [27,28]. In this study, the chimeric protein P97R1P46P42 was expressed by the baculovirus expression system by fusing with the molecular adjuvant CD40L. The chimeric fusion protein was evaluated in the mouse model to see if CD40L could potentiate the immune responses.

## 2. Materials and Methods

### 2.1. Cells Lines, Plasmids and Viruses

*E. coli* TG1, *E. coli* DH10Bac strain, and *E. coli* TG1 pFastBac Dual (pFBD) and *E. coli* Top10 pUC57-SPgp64-p97r1p46p42-mCherry-TMDgp64 as well as Sf9 cells (*Spodoptera frugiperda* cell, grass-craving noctuid moth cell) were kept in our laboratory.

### 2.2. Selection of Coding Sequences and Gene Design and Construction of the Baculovirus Expression Vector

Recombinant Mhp P97R1P46P42 chimeric protein has been previously expressed in our laboratory using a prokaryotic expression system and an insect baculovirus surface display system [27,28]. In the present study, to obtain the recombinant plasmid pFBD-P97R1P46P42, the optimized P97R1P46P42 gene sequence was inserted between the *Sac*I and *Xba*I restriction enzyme sites of the pFastBac Dual vector.

The gene sequence of CD40L was retrieved from the NCBI database (accession number: NM214126). Codon optimization was performed on its exon sequence. To increase the immunogenicity of the antigen, the trimeric motif of T4 phage fibronectin (GYIPEAPRDGQAYVRKDGEWVLLSTFL) was added to the design of other studies [29,34,35]. The fragment was attached to the N-terminus with a GGPGS junction and a 6× His tag was added at the C-terminus. The recombinant plasmid pFBD-P97R1P46P42-CD40L was obtained by inserting this CD40L into the plasmid pFBD-P97R1P46P42 between the restriction sites of *Xho*I and *Xba*I.

### 2.3. Preparation of Recombinant Baculovirus Plasmids

The recombinant plasmids pFBD-P97R1P46P42 and pFBD-P97R1P46P42-CD40L were transformed into *E. coli* DH10Bac competent cells. Recombinant DH10Bac colonies were picked after blue-white screening, and baculovirus plasmid (Bacmid) was extracted and identified.

### 2.4. Protein Expression, Purification and Identification

Sf9 cells were diluted to 4 × 10^5^ cells/mL and then resuspended in 12 mL of Sf-900™ II medium (Gibco, Grand Island, NY, USA). Cells were re-plated to a six-well plate 2 mL per well, mixed well, and allowed to grow overnight at 27 °C. When the cell confluence reached 70–80%, recombinant bacmid was transfected into Sf9 cells according to the instructions for FuGENE^®^ 6 Transfection Reagent (Promega, Madison, WI, USA). Cells were cultured at 27 °C and observed daily. Four days later, the supernatant containing the virus was collected and marked as recombinant baculovirus generation P0. The Sf9 cells at a cell density of 2.0 × 10^6^ cells/mL were infected using 0.5% P0 baculovirus. The cells were cultured for 72 h at 110 rpm, 27 °C. The supernatant was collected by centrifugation to obtain the generation of P1 baculovirus. The amplification was repeated one more time to obtain the generation of P2 baculovirus.

P2 baculoviruses were subjected to viral amplification in Sf9 cells at 5%. After 72 h, the cells were lysed and cytosol fraction was collected for purification of the chimeric proteins using a nickel column (His60 Ni Superflow Resin, Clontech, Mountain View, CA, USA). The chimeric proteins were analyzed using 12% SDS-PAGE gels and Western blotting (WB). Primary antibodies were laboratory-prepared rabbit polyclonal antibodies to rP97, rP42, and rP46 proteins (1:3000 dilution). Horseradish peroxidase (HRP)-conjugated anti-His monoclonal antibody (Proteintech, Rosemont, IL, USA) was used at 1:3000 dilution. Secondary antibodies were probed with HRP-labeled goat anti-rabbit IgG (H + L) (1:1000 dilution; Beyotime, Shanghai, China).

### 2.5. Immunization of Mice

Forty 6–8-week-old SPF-grade female BALB/c mice, with a body weight of 20 g ± 2 g, were randomly assigned to five groups: (1) PBS group, (2) rP97R1P46P42 experimental group (P97R1P46P42 group), (3) rP97R1P46P42 plus ISA 201 adjuvant emulsion experimental group (ISA 201 group), (4) rP97R1P46P42-CD40L experimental group (CD40L group), and (5) commercial vaccine group (CV group). Each group of 8 mice was randomly labeled 1–8 with picric acid to facilitate subsequent experiments.

The mice were immunized by the dorsal subcutaneous multiple injections and then kept in individually ventilated cages (IVCs). Immunization was carried out three times on days 0, 14 and 28. Each mouse received 200 μL of sterile phosphate-buffered saline (PBS group), the chimeric protein (0.2 mg/mL, P97R1P46P42 group), fully emulsified P97R1P46P42 immunogen (0.2 mg/mL, Seppic ISA 201 group), P97R1P46P42-CD40L protein solution diluted to 0.2 mg/mL (CD40L group) or Rebis-Wang^®^ inactivated *Mycoplasma hyopneumoniae* swine vaccine (strain *p*-5722-3, CV group).

Blood was collected from the orbital venous plexus on days 0, 7, 14 and 21, and from the submandibular vein on days 28, 35 and 42 from four mice in each group. The blood samples were at 4 °C overnight. Serum samples were separated on the following day by centrifugation at 4 °C, 3000 rpm for 10 min and stored at −80 °C. Cellular immunity was detected by splenic lymphocyte proliferation assay on three mice in each group at days 35 and 42.

### 2.6. Analysis of Antibody Responses

Based on previous studies, serum samples collected from mice were tested for antigen–antibody responses to the antigenic protein P97R1P46P42 by indirect ELISA [28]. Subunit protein antigen (rP97R1P46P42 at a concentration of 1 µg/mL, 100 µL per well) was coated on microtitre plates at 4 °C overnight. A volume of 100 μL of the mouse serum samples, diluted using sample diluent at a ratio of 1:100, was transferred to the coated wells and incubated at 37 °C for 1 h. The plate wells were then washed and HRP-coupled goat anti-mouse IgG (H + L) (dilution 1:2000, Beyotime) was added and incubated at 37 °C for 1 h. Tetramethylbenzidine was used as the substrate and absorbance was measured at an optical density (OD) of 450 nm using the microplate reader (Bio-Tek, Winooski, VT, USA).

### 2.7. Lymphocyte Proliferation Test

On days 35 and 42, after blood collection, mice were euthanized and spleen samples were collected. Splenic lymphocytes were isolated using a Mouse Lymphocyte Separation Medium (DAKEWE, Shenzhen, China), and specific experimental procedures were performed according to the instructions. Isolated splenic lymphocytes were counted. The cell density was adjusted to 4.5 × 10^6^ cells/mL and inoculated in triplicate into 96-well cell culture plates (12 replicate wells for each sample). In detail, 100 µL RPMI-1640 (Gibco) containing 20 µg rMhp-P97R1P46P42 was used as a stimulator, and RPMI-1640 and concanavalin A (20 µg/mL, Solarbio, Beijing, China) were added as negative and positive controls, respectively. The cells were incubated at 37 °C for 42 h in a cell culture incubator. Twenty μL of MTT stock solution (5 mg/mL, Solarbio) was added to each well, and incubated for 4 h at 37 °C in a cell culture incubator. The supernatants were then discarded and 100 μL of DMSO (Macklin, Shanghai, China) was added to each well to dissolve the formazan. The plates were then shaken and mixed in a shaker for 5 min, followed by OD measurement at 490 nm. Stimulation values were calculated as follows: Stimulation (%) = mean OD of antigen-stimulated cells (addition of rP97R1, rP46, and rP42)/mean OD of unstimulated cells (addition of RPMI-1640)/stimulation of mean OD of cells by concanavalin A.

### 2.8. Detection of Serum Cytokine Levels

Cytokine IL-4, as well as IFN-γ levels, were measured in mouse serum samples on days 35 and 42 according to the instructions of the Mouse Interleukin 4 (IL-4) ELISA Kit (MEIMIAN, Yancheng, China, MM-0165M1) and the Mouse Interferon-gamma (IFN-γ) ELISA Kit (MEIMIAN, MM-0182M1). The kits are based on the double-antibody sandwich ELISA principle. Briefly, the test samples with appropriate dilutions and the reference standards were transferred into the wells of precoated plates and incubated for 30 min at 37 °C, followed by 5X washes. HRP-conjugated antibodies were then added and the plates were incubated again for 30 min at 37 °C. Tetramethylbenzidine as the substrate solution was added after washing and OD450nm was measured after 10 min at 37 °C.

### 2.9. Statistical Analysis

All data are shown as mean ± SD of six mice (antibody responses) and three mice (lymphocyte proliferation, IL-4 and IFN-γ), and were analyzed by one-way ANOVA for between-group comparisons or by two-way ANOVA for bivariate effects of vaccination and timing on GraphPad Prism 8.0. Bonferroni correction was used to adjust the *p* values.

## 3. Results

### 3.1. Vector Construction, Chimeric Protein Expression and Identification by Western Blotting

**Figure 1:**
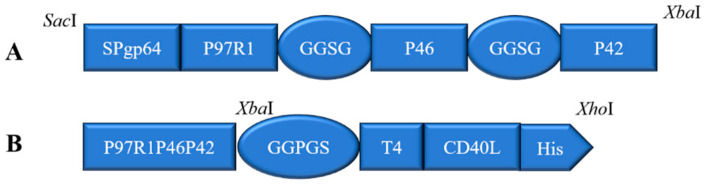
Map of target recombinant protein fragments (**A**) P97R1P46P42; (**B**) P97R1P46P42-CD40L.

**Figure 2:**
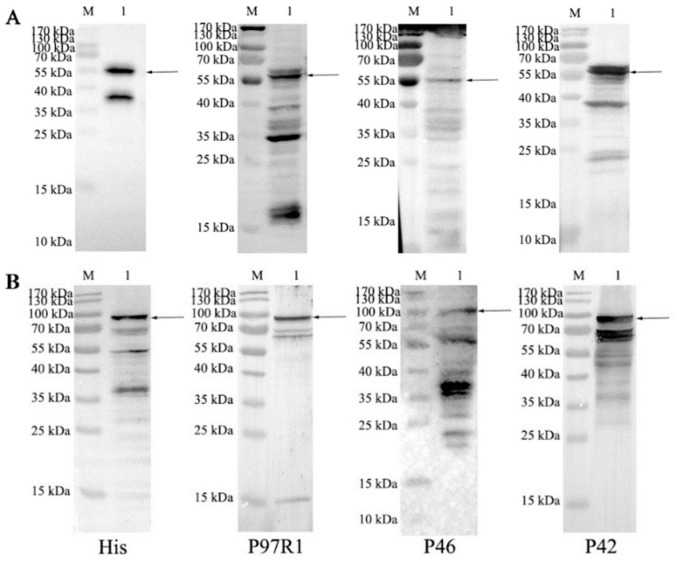
Identification of expressed recombinant proteins by Western blotting. M: protein molecular weight standard; (**A**): recombinant protein P97R1P46P42; (**B**): recombinant protein P97R1P46P42-CD40L; His: probed with anti-His monoclonal antibody; P97R1, P46 and P42: probed with rabbit polyclonal antibodies prepared from rP97R1, rP46 and rP42, respectively.

Two recombinant baculovirus plasmids pFBD-P97R1P46P42 (Figure 1A), and pFBD-P97R1P46P42-CD40L (Figure 1B) were constructed and obtained by blue-white screening. They were further identified by PCR using primers PpH_Forward and Psv40_Reverse, and universal primer M13, and by sequencing. The recombinant plasmids were transfected into sf9 cells for protein expression. The cells were collected and the expressed recombinant proteins were identified by Western blotting. Figure 2 shows that the recombinant proteins P97R1P46P42 and P97R1P46P42-CD40L were successfully expressed as shown by the appearance of expected molecular sizes using the anti-p97r1, anti-p46, anti-p42, and anti-6×His antibodies.

### 3.2. Purification and Identification of Chimeric Proteins

**Figure 3:**
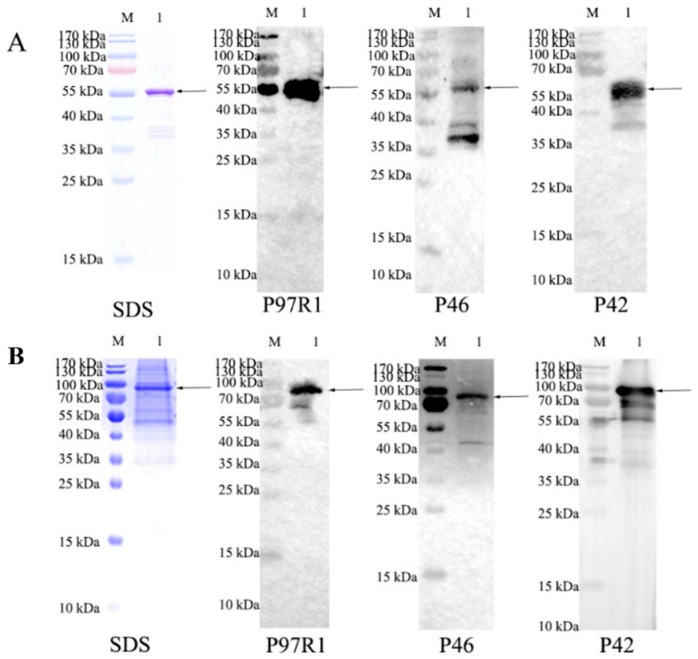
Identification of purified recombinant proteins by Western blotting. M: protein molecular weight standard; (**A**): recombinant protein P97R1P46P42; (**B**): recombinant protein P97R1P46P42-CD40L; SDS: SDS-PAGE; P97R1: P97R1, P46 and P42: probed with rabbit polyclonal antibodies prepared from rP97R1, rP46 and rP42, respectively.

After nickel column purification, the purified recombinant proteins were identified by Western blot using three primary antibodies, anti-p97r1, anti-p46, and anti-p42 (Figure 3). A band at the expected size proves that the purified protein is the target protein. The purified proteins were assayed for concentration and the proteins were preserved for later animal immunological experiments.

### 3.3. Immunogenicity Assessment of the Chimeric Proteins

**Figure 4:**
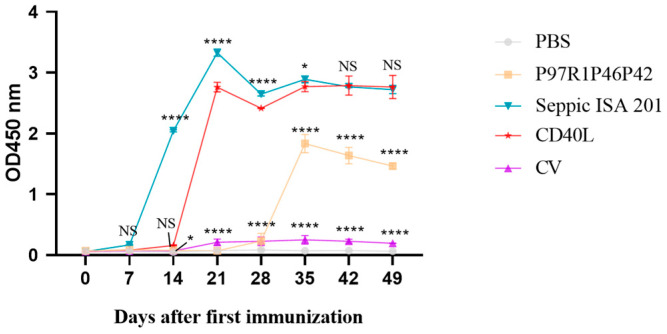
The antibody responses in sera from different groups of mice. NS, no significant difference * *p* < 0.05, **** *p* < 0.0001; where **** *p* < 0.0001 is considered an extremely significant statistical difference when compared with the CD40L group. Two-way ANOVA and Bonferroni correction were used for significant analysis and multiple comparisons, respectively.

To investigate whether CD40L could enhance the induction of humoral immune response in mice by rP97R1P46P42 chimeric proteins, we detected the specific antibody responses in mouse sera by using an indirect ELISA (Figure 4). Mice in the CD40L group began to have a surge in specific IgG antibodies on day 14, which was significantly higher than those in the P97R1P46P42 protein group and all other groups including the CV group from day 21 and thereafter. From day 14 to 28, the antibody responses in the Seppic ISA 201 group were more prominent than in the CD40L group. However, there was a minor (*p* < 0.05) or no significant (*p* > 0.05) difference in the antibody responses between the CD40L group and the ISA 201 group.

**Figure 5:**
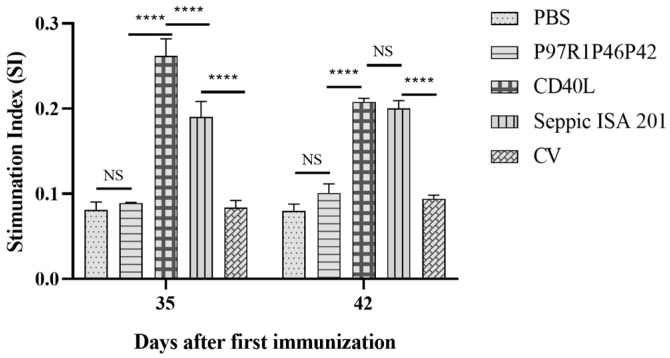
Chimeric P97R1P42P46-CD40L fusion protein enhanced the proliferation of spleen lymphocytes from immunized mice. NS, no significant difference, **** *p* < 0.0001; where **** *p* < 0.0001 is an extremely significant statistical difference. One-way ANOVA and Bonferroni correction were used for significant analysis and multiple comparisons, respectively.

To investigate whether CD40L could enhance the cellular immune potency of rP97R1P42P46 chimeric protein in mice, the lymphocyte proliferation assay was used to test the stimulation index (SI) of the splenic lymphocytes of each group of mice on days 35 and 42 in response to the protein P97R1P42P46 (Figure 5). At both time points of day 35 and 48, the SIs of the CD40L and ISA 201 groups were significantly higher than the P97R1P46P42 and CV groups (*p* < 0.0001).

**Figure 6:**
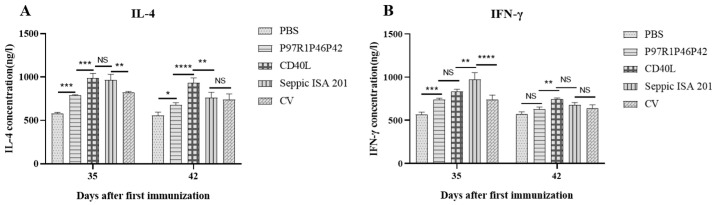
IL-4 and IFN-γ levels in sera of mice from different groups by indirect ELISA. (**A**): IL-4 and (**B**): IFN-γ. NS, no significant difference, * *p* < 0.05, ** *p* < 0.01, *** *p* < 0.001, **** *p* < 0.0001; where *** *p* < 0.001 is an extremely significant statistical difference. One-way ANOVA and Bonferroni correction were used for significant analysis and multiple comparisons, respectively.

IL-4 and IFN-γ are representative cytokines for Th2 and Th1 cells, respectively. IL-4 and IFN-γ levels in the sera of mice on days 35 and 42 were performed by using commercial indirect ELISA kits. Figure 6 shows that CD40L fusion significantly increased the production of IL-4 than the P97R1P42P46 protein alone at both time points, though the P97R1P42P46 protein could induce IL-4 as compared to the PBS group (Figure 6A). The IL-4 level in the CD40L group was similar to or even higher than the Seppic ISA 201. Both the P97R1P42P46 protein and its fusion with CD40L increased the IFN-γ content as compared to the PBS group, and the fusion protein tended to have higher IFN-γ than the P97R1P42P46 protein, though not statistically different at day 35, but markedly different at day 42 (Figure 6B). The mice receiving chimeric protein with Seppic ISA 201 emulsion exhibited the highest IFN-γ level on day 35 while those receiving CD40L-fused protein showed the highest on day 42.

## 4. Discussion

Currently, the prevention and treatment of mycoplasmal pneumonia in swine is mainly through vaccination [36]. Although many vaccines have been developed worldwide against the disease, full protection is barely reached. Genetically engineered vaccines, despite their many advantages, have low immunogenicity [37]. Immunomodulatory molecule-like adjuvants act on the immune system through the target receptors, enhancing differentiation of relevant immune cells, and can be expressed in fusion with antigens to enhance immune responses. Such adjuvants will only activate antigen-presenting cells that come into contact with the antigens, thus lowering the dose of the antigens and reducing the possibility of adverse effects [38,39,40]. They usually have better safety profiles and are more suitable for improving the immune responses of genetically engineered subunit vaccines.

CD40L affects hematopoiesis as well as the development and migration of neutrophils and can mediate various immune responses and inflammatory reactions [41]. It is one of the strongest inducers of Th1 responses, stimulating both innate and acquired immunity, and is a good molecular adjuvant [42]. CD40L) was fused to SARS-CoV-2 spiking protein as a molecular adjuvant and targeting ligand and the fusion protein induced significant neutralizing antibodies, improved the immune response and prevented infection in the nasal turbinates and lungs [43]. CD40L might function as an antigen-targeting molecule and adjuvant, it, when expressed in combination with the syncytial viral protein F, can effectively induce neutralizing antibodies and memory CD8T cells, thereby protecting animals against RSV infection without causing respiratory illness [34]. Huang et al. demonstrated that duck CD40L showed a surprising ability to greatly increase the titers of neutralizing antibodies and the generation of IFN-γ evoked by a DNA vaccine encoding the Tembusu virus, an avian flavivirus, and improved viral clearance of the prM-E DNA vaccination in ducks [44].

Preliminary studies in our laboratory revealed that the recombinant Mhp P97R1P46P42 chimeric antigen has good immunogenicity in both mouse and piglet models and can be used as a candidate antigen for the genetically engineered subunit vaccine against MPS [27,28]. To further enhance the immunogenicity of this vaccine candidate, CD40L was expressed in fusion with the P97R1P46P42 protein in this study. Immunization in the murine model revealed that the molecular adjuvant CD40L could enhance the humoral and cellular immune responses induced by rP97R1P46P42 protein in BALB/c mice, in levels similar to the Seppic ISA201, but superior to the conventional vaccine.

Figure 4 clearly indicates that CD40L could markedly increase the level of specific antibodies in sera from immunized mice as compared with the non-fused P97R1P46P42 protein. The levels of IgG antibodies did not differ significantly in the ISA 201 and CD40L groups on days 42 and 49, indicating that the adjuvant-enhanced induction of humoral immune response was comparable to the commercial ISA 201 emulsion in terms of the persistence of the effect in the later stages. Overall, this result is similar to other studies in enhancing the humoral immune response to the vaccines [34,45,46]. Li et al. demonstrated that CD40L enhanced the humoral immune response and the stability of immunogenicity of the PCV2 adenovirus vaccine [45]. CD40L fusion also enhanced the cellular immune response of the P97R1P46P42 chimeric protein in mice better than ISA 201 (Figure 5), and favored the Th2 immune response shown as marked elevation of IL-4 (Th2 hallmark) (Figure 6A). Detection of IFN-γ (major Th1 cytokine) levels in mouse sera showed (Figure 6B) that CD40L could enhance rP97R1P46P42 protein-induced Th1 responses at day 35, but not at a later stage.

Our work provided further evidence that CD40L adjuvant could promote both humoral and cellular immune responses to mycoplasmal antigens, as seen with other antigenic components, such as *Eimeria tenella* protein 1 by chicken CD40L [47]. In a study with human immunodeficiency virus-1 (HIV-1) canarypox vaccinations, CD40L enhanced Th1 cytokine production in response to vaccine antigens as well as splenocyte proliferation, but did not improve humoral immunity [48]. In other studies, CD40L enhances both humoral and cellular immune responses [29,43,49]. For example, in an influenza A vaccine study, HA2-specific T cell responses, HA2-specific mucosal IgA, and serum IgG levels were all increased by using CD40L as a molecular adjuvant [29].

This study clearly indicates that the Mhp P97R1P46P42 chimeric protein from the baculovirus expression system is a promising subunit vaccine candidate against MPS upon fusing with CD40L as the molecular adjuvant. The CD40L-fusion antigen exhibited enhanced immunogenicity in mice. A number of early studies have shown that the immune responses to the Mhp antigens were similar in mice and pigs [27,50,51]. There are also quite a number of studies related to immunogenicity of Mhp vaccines or vaccine candidates in the murine model as shown in the review paper [52] as well as in a recent publication by Santos et al. [53]. However, the murine model is not suitable for studying the protective effects of vaccines, including subunit vaccines, against challenges with pathogenic Mhp because the mouse is not a susceptible host to this particular pathogen. In this regard, further research is warranted to examine the protective efficacy of CD40L-fused Mhp P97R1P46P42 chimeric protein in the pig model against challenges with Mhp infection.

## 5. Conclusions

The molecular adjuvant CD40L enhances the humoral and cellular immune responses induced by the Mhp recombinant protein rP97R1P46P42 in the mouse model. These results support further efficacy testing of the CD40L-fused chimeric Mhp protein as an MPS subunit vaccine candidate in pigs in response to challenges with pathogenic *Mycoplasma hyopneumoniae* strain(s).

## Data Availability

The original contributions presented in this study are included in the article. Further inquiries can be directed to the corresponding author.
